# Generative cerebral vasculature visualization using spatial transcriptomic data

**DOI:** 10.1038/s41598-026-46455-4

**Published:** 2026-03-31

**Authors:** Ingrid Berg, Jiqing Wu, Viktor H. Koelzer

**Affiliations:** 1 Department of Pathology and Molecular Pathology, University Hospital Zurich, University of Zurich, Zurich, Switzerland; 2 Department of Biomedical Engineering, University of Basel, Basel, Switzerland; 3 Institute of Medical Genetics and Pathology, University Hospital Basel, Basel, Switzerland

**Keywords:** Spatial transcriptomics, Generative modeling, Cerebral vasculature, Computational biology and bioinformatics, Neuroscience, Systems biology

## Abstract

The brain is sustained by an intricate vascular network that provides a continuous supply of nutrients and oxygen essential for its function. Understanding the structural variability and region-specific function of this neurovascular system is essential for interpreting neurological alterations in disease models. Leveraging the spatial mRNA-guided generative model Tera-MIND (simulating **Tera**-scale **M**ouse bra**IN**s using a patch-based and boundary-aware **D**iffusion model), we propose to predict the spatial organization of brain vasculature by the co-expression patterns of *Cldn5* and *Acta2* genes and their learned attention map at cell-level resolution. These findings demonstrate the capability of generative AI models trained on single-cell spatial transcriptomic data to capture biologically meaningful higher-order structures. They highlight the potential of such models as in silico systems - GenAI-based simulations that generate realistic representations of biological architecture from spatial molecular data - for high-throughput exploration of vascular function and its dysregulation in neurological disorders. Taken together, our approach repurposes existing spatial transcriptomics datasets to derive new spatial insights into vascular organization, without the need for additional tissue processing.

**Supplementary Information:**

The online version contains supplementary material available at 10.1038/s41598-026-46455-4.

## Introduction

Neurons rely on continuous blood flow due to their high energy requirements and low capacity for energy storage. Even small disruptions in blood flow can impair neuronal function and survival. Neuronal activity, in turn, modulates blood flow, and several neurological diseases involve vascular dysfunction^1^. Mapping the cerebrovascular network is challenging due to the large variety of blood vessel calibers, from capillaries to large vessels, in a complex 3D structure. However, brain-wide vascular maps are fundamental for understanding how vascular network organization supports neural function and how changes in this system contribute to disease^1^. Recent developments in generative artificial intelligence (GenAI) have supported the in-silico synthesis of realistic biological structures spanning different spatial scales. With complex datasets such as biomedical images and spatial transcriptomics as inputs, GenAI models can learn to infer tissue microanatomy and simulate morphological transitions, establishing in silico modeling of whole organisms^2^. In the recent Tera-MIND study, the authors proposed a novel GenAI approach that can capture spatial associations between gene expression and histological image patterns, exemplified by key pathways involved in glutamatergic and dopaminergic neuronal systems^3^.

In this application study, we further investigate the potential of generative AI to model biologically meaningful higher-order structures using single-cell spatial transcriptomics data. We build directly on the previously introduced Tera-MIND generative framework and apply it to a biological domain that was not explored in the original study: the visualization of vasculature-associated structural patterns from spatial gene expression. This work demonstrates how a generative, gene-conditioned model can be used to explore higher-order vascular organization, highlighting a new use case for multimodal diffusion models in spatial transcriptomics. Rather than introducing methodological changes to Tera-MIND, our contribution lies in showing that the framework enables biologically interpretable, gene-guided visualization of cerebrovascular architecture.

## Results

**Fig. 1:**
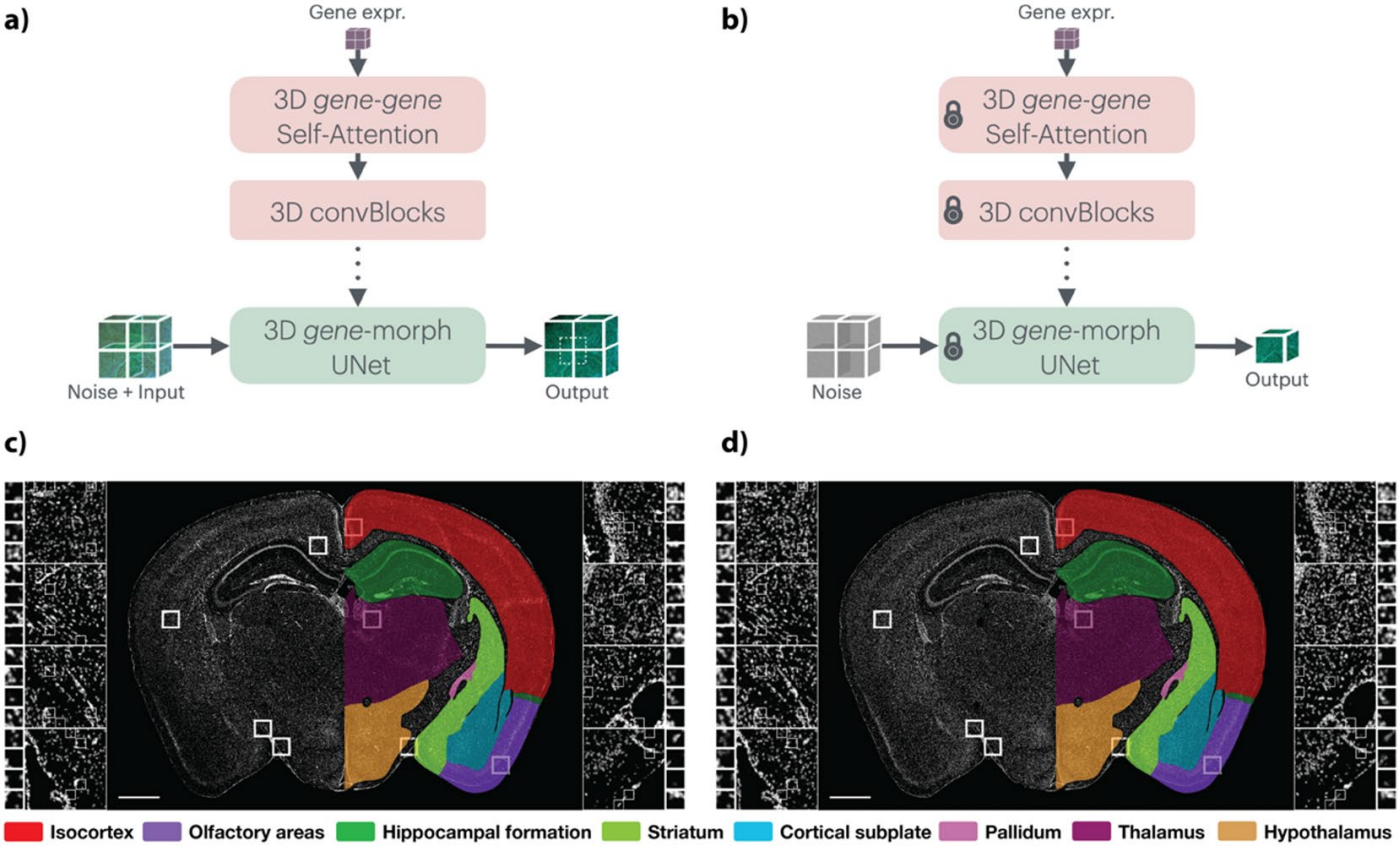
Model training and testing with representative ground-truth and generated DAPI-stained sections. (**a**) Illustration of patch-based diffusion model training. Spatial mRNA coordinates from two mouse brains are converted into 3D gene-expression arrays and paired with matched DAPI and PolyT image tiles. A patch-based 3D diffusion model is trained, in which a 3D gene-gene self-attention block learns local gene co-occurrence and injects these embeddings into a 3D UNet that denoises noisy DAPI/PolyT patches into clean patterns, with a boundary-aware path enforcing consistency between neighboring patches. (**b**) Illustration of model test/inference on an unseen brain. Only spatial mRNA data are provided. Tera-MIND synthesizes corresponding DAPI/PolyT patches, which are tiled to obtain full coronal sections and volumes. (**c**) Image showing the ground truth of a DAPI staining of a coronal section of the mouse brain, including color labels of major brain regions indicated for anatomical reference. (**d**) Image showing the generated version of the DAPI staining for the same section, showing preservation of large-scale anatomical organization. (**c**,**d**) Length of scale bar (white, bottom left corner) is 1 mm. Mouse brain ID: 638850.

**Fig. 2:**
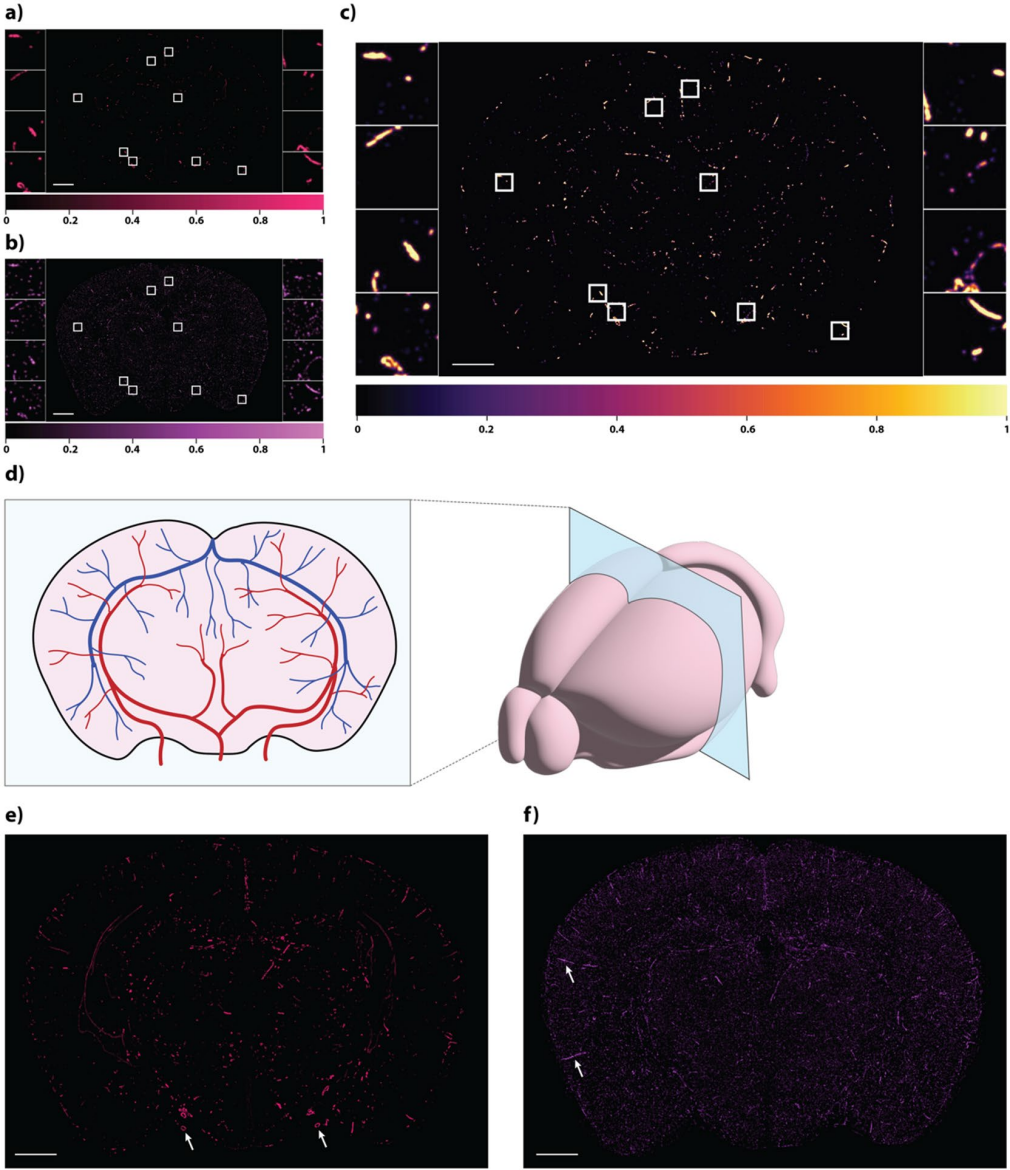
Generative modeling of cerebrovascular architecture from spatial transcriptomic data. (**a**) Generated image showing *Acta2* expression in a coronal brain section consistent with localization to vascular smooth muscle cells. (**b**) Generated image showing *Cldn5* expression in a coronal brain section, consistent with localization to endothelial tight-junctions which are relevant for blood-brain barrier integrity. (**c**) Visualization of *Cldn5*-*Acta2* gene-gene attention, highlighting regions where the model identifies co-occurring vascular marker patterns. In Tera-MIND, this attention signal summarizes local co-expression of *Cldn5* and *Acta2* into a molecular context that conditions the diffusion model and helps guide the emergence of vessel-like structures in regions where these markers align. (**a**–**c**) Signal intensity colorbar indicated below figure. (**d**) Simplified schematic illustration of typical vasculature distribution for the corresponding coronal level, consistent with published vascular atlases (e.g. Xiong et al.^6^). (**e**) Generated *Acta2* expression patterns visualized in an overlay of 4 consecutive coronal sections. White arrows indicate expression patterns resembling cross-sections of large blood vessels, consistent with major arterial territories^6^. (**f**) Generated *Cldn5* expression patterns visualized in an overlay of 4 consecutive coronal sections. White arrows indicate expression patterns resembling longitudinally sectioned blood vessels, consistent with known vascular anatomy^5^. (**a**–**f**) Length of scale bar (white, bottom left corner) is 1 mm. Mouse brain ID: 638850.

With Tera-MIND, we can visualize an integrated model of gene expression in the neurovascular context. Specifically, we apply the Tera-MIND approach (Fig. 1a and b) to generate high-resolution, spatially-resolved visualizations of cerebral microvascular structures, creating a virtual representation of murine brains (exemplified in Fig. 1c and d) with projected microvasculature. This enables the spatial analysis of gene expression patterns in relation to vascular architecture and neurovascular function (Fig. 2a-f). In this application study, we focus on two vasculature-associated genes for which spatial transcriptomic information was available in all three mouse brain data sets: *Cldn5* and *Acta2*. Both *Cldn5* and *Acta2* expression play key roles in the function of the neurovascular unit, and dysfunction of these genes is associated with various neurological diseases.

### 3D modeling of claudin-5 expression in the murine brain

*Cldn5*, a gene encoding the endothelial tight-junction protein claudin-5, is critical for blood-brain barrier (BBB) integrity and is known to exhibit highest expression in brain capillaries and small venules^4^. Using the Tera-MIND model, we predicted *Cldn5* expression across the brain and observed spatial patterns that correspond closely to known vascular anatomy (Fig. 2b). Notably, regions with longitudinally sectioned vessels, such as those extending from the cortex into deeper brain layers^5^, exhibited strong and continuous predicted *Cldn5* signal (Fig. 2f, representative merged image of four adjacent sections), consistent with the expected localization of capillary-rich zones. These findings demonstrate that the model successfully captures biologically meaningful vascular features relevant to BBB-associated gene expression.

### 3D modeling of alpha smooth muscle actin expression

*Acta2*, which encodes alpha smooth muscle actin, is predominantly expressed in vascular smooth muscle cells and marks arterial structures, particularly larger arteries^1^. Tera-MIND predictions of *Acta2* expression localized to regions consistent with major arterial territories, including the ventral surface of the brain where larger arteries are anatomically expected^6^ (Fig. 2a and e). To explore spatial interactions between vascular components, we jointly analyzed *Acta2* and *Cldn5* expression using gene-gene attention mapping. This analysis revealed co-localized expression within shared vascular segments (Fig. 2c), suggesting that the model accurately captures complementary features of the vascular hierarchy - from endothelial markers of the blood-brain barrier to smooth muscle-associated arterial identity.

### Vascular network inference

Within Tera-MIND, the gene-gene attention block summarizes local co-expression of *Cldn5* and *Acta2* into a molecular context that conditions the diffusion model, allowing the inferred vascular-like patterns to follow vessel-like trajectories even when individual mRNA molecules are sparsely sampled. To infer the vascular architecture of the healthy mouse brain, we applied the model to consecutive 2D spatial transcriptomics sections, focusing on genes associated with the neurovascular unit. The resulting 3D-integrated predictions enabled visualization of vascular continuity and organization across anatomical regions. Predicted *Cldn5* (Fig. 2f) and *Acta2* (Fig. 2e) expression highlight endothelial and smooth muscle components, respectively. These predictions are reproducible in all three mouse brains analyzed (Fig. 2e-f and Suppl. Figure 1), align with known vascular structures^6^ (schematically illustrated in Fig. 2d), and support downstream analyses of spatial gene expression in the context of vascular integrity.

### Correlation analysis across whole brain sections

**Fig. 3:**
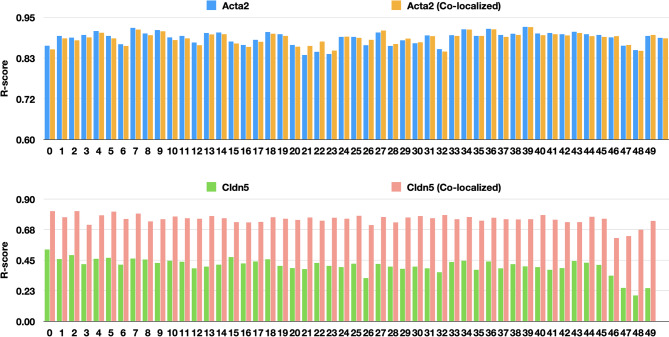
Quantitative validation of vascular prediction consistency. (Top): R-scores for *Acta2* expression vs. model attention for all regions and the regions where both genes are co-localized across 50 mouse brain slices. (Bottom): R-scores for *Cldn5* expression vs. model attention for all regions and the regions where both genes are co-localized across 50 mouse brain slices.

To quantify the consistency of our model across the 50 P56 mouse brain slices, we calculated the Pearson correlation coefficient (R) between the model’s attention maps and the spatial expression of key vascular markers, *Acta2* and *Cldn5*. **High Inter-sample Consistency**: As shown in Fig. 3, *Acta2* demonstrates a robust and high correlation with the predicted vessel structures across all 50 samples, maintaining an average R-score of 0.89. **Synergistic Predictive Power**: While *Cldn5* alone exhibited a moderate correlation, we observed a significant increase in predictive accuracy when analyzing regions where *Acta2* and *Cldn5* co-localize. This suggests that the model effectively integrates signals from multiple markers to define vascular architecture.

## Discussion

In this study, we present a generative, spatial gene-informed in silico model of the murine cerebral vasculature, modeled at teravoxel scale from single-cell spatial transcriptomics data. By leveraging vascular marker genes, the model enables the formation of coherent, spatially-continuous vascular structures that reflect known anatomical and molecular features reproducibly in all three mouse brains analyzed. This virtual representation supports high-resolution spatial analysis of gene expression in relation to vascular patterns, offering a new framework to study neurovascular organization and function in healthy tissue. Importantly, this work establishes a scalable approach for modeling higher-order tissue structures from single-cell spatial data within their native spatial context, enabling the investigation of gene expression at cellular resolution.

While the original Tera-MIND study focused primarily on neuronal patterns, the present work demonstrates a biological application that was not explored previously: visualization of vasculature-associated structural patterns. This represents a conceptual extension of the Tera-MIND framework to vascular systems biology and highlights its usefulness for interrogating higher-order tissue organization beyond the neuronal domain.

Although advanced imaging methods such as light sheet or serial two-photon microscopy provide high-resolution vascular compartment visualization, they remain time-consuming and depend on labor-intensive protocols. In contrast, by leveraging the modern high-throughput GenAI approach and reusing spatial transcriptomics datasets, our approach extends their utility by enabling structural analyses, including gene-informed generative visualizations of vascular marker patterns, cell-cell interactions and co-localization. Thus, Tera-MIND provides a complementary avenue for extracting structural insights, extending rather than replacing imaging-based approaches.

Looking ahead, this modeling framework could be extended to incorporate additional modalities and conditions, enabling more comprehensive 3D simulations of tissue organization. Integration with complementary histological, imaging, and genomic datasets may enhance the model’s capacity to capture structural and functional variability. While this study focuses on healthy brain tissue, the generative approach lays the foundation for future in silico exploration of disease-associated perturbations, such as blood-brain barrier dysfunction, once appropriate spatial transcriptomic datasets from disease models become available.

### Limitation

A key aspect of our approach lies in its multi-scale ability for interpretable image prediction, enabling detailed visualization of vascular structures from gene expression data. However, a limitation of the current study is that 3D modeling is based on ground truth data with non-contiguous 2D sections with 200 micron gaps^7^. This inherent sparsity in the ground truth data introduces challenges for accurate 3D vascular network inference, potentially leading to discontinuities or artifacts. Because the model relies on sparse single-molecule input and non-contiguous section sampling, the resulting gene-guided vascular-like patterns should be interpreted as a probabilistic inference rather than an exact reconstruction. The spatial context used during training allows Tera-MIND to align large-scale vascular territories across slices, whereas fine-scale topology cannot be deterministically recovered across 200 micron section gaps. In addition, the inferred vascular-like patterns depend on the local availability of vasculature-associated transcripts; the model can interpolate across moderately sparse regions but cannot recover vessel structures in areas where characteristic marker signal is absent. Moreover, while the inferred vascular-like patterns show anatomically plausible regional organization that reflects the known distribution of endothelial (*Cldn5*) and smooth muscle (*Acta2*) markers, the current data do not permit validation of fine-scale vascular topology. As such, our results should be interpreted as gene-guided visualizations of vascular territories rather than reconstructions of detailed vascular architecture. One promising strategy to address the limitation of non-contiguous 2D sections has been shown by Turos et al. with X-Pression, which combines single 2D spatial transcriptomics sections with micro-CT imaging to enable full 3D representation of gene expression patterns at the tissue level, eliminating the need for serial sectioning of entire organs^8^.

Further, our GenAI approach was developed using spatial transcriptomic data that lack quantitative validation against independent imaging data generated with orthogonal methodologies (e.g., light-sheet microscopy or µCT angiography). This limitation primarily arises from the scarcity of datasets profiled with these different technologies on the same or consecutive brain slices, which would enable reliable quantitative comparison. Nonetheless, this data availability issue may be alleviated by the increasing emergence of multi-omics mouse brain cohorts, which could help bridge this validation gap in future work.

### Marker selection

**Fig. 4:**
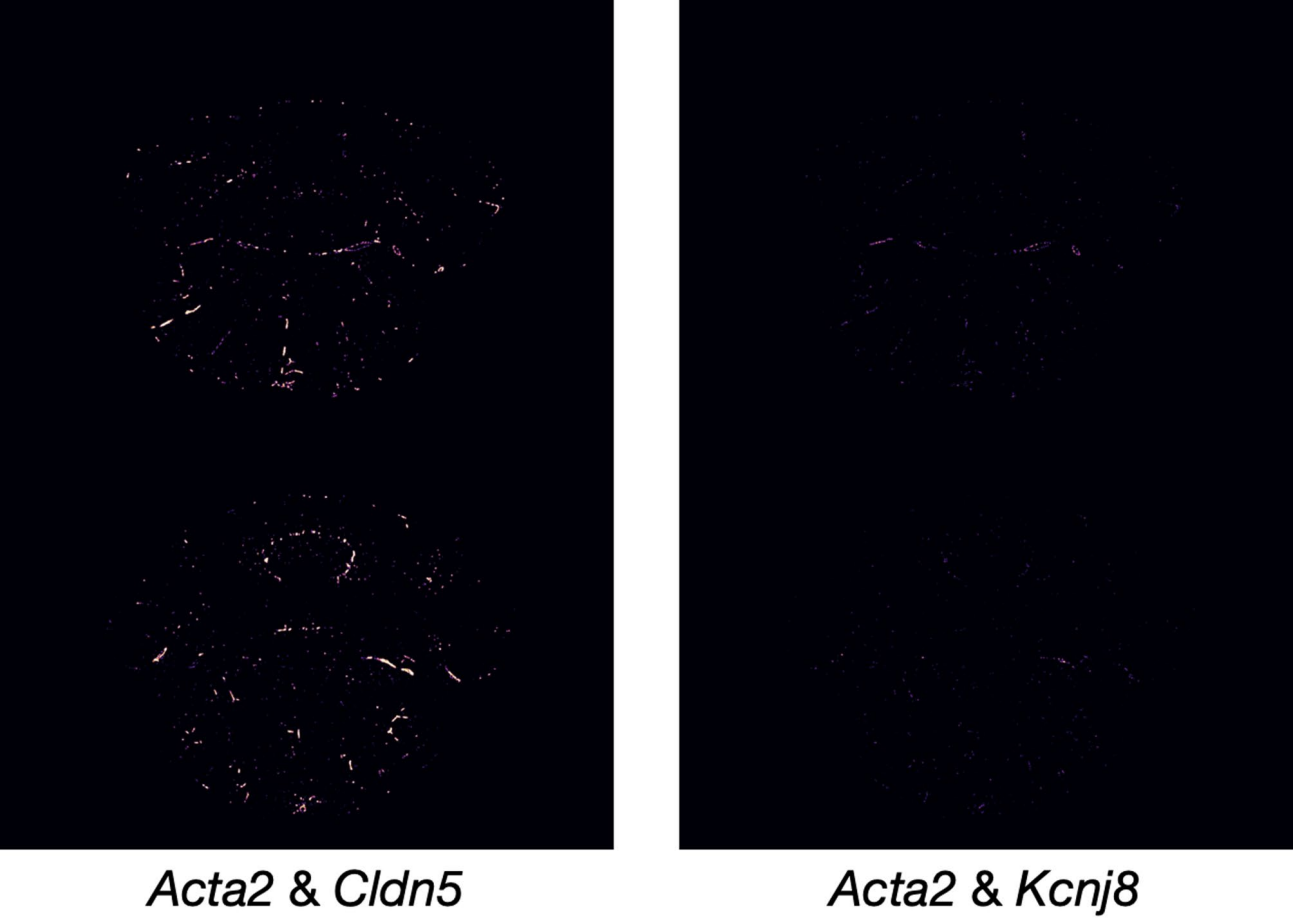
The side-by-side comparison for the generative modeling of cerebrovascular architecture using two different pairs of genes. (Left): *Acta2* and *Cldn5* attention maps shown for brain sections from two different anatomical regions (top and bottom of image). (Right): *Acta2* and *Kcnj8* attention maps shown for brain sections from two different anatomical regions (top and bottom of image).

In this proof-of-concept study, we focused on *Cldn5* and *Acta2* because they represent two major and well-characterized vascular compartments: endothelial cells of the blood-brain barrier and smooth muscle vascular structures. Also, they were consistently measured across all three mouse brains used for training and testing. While this minimal marker set effectively highlights capillary-rich and arterial territories, it cannot capture the broader diversity of vascular cell types. Moreover, vasculature-associated genes that were not uniformly represented across the three available datasets could not be included, limiting the granularity of vascular biology that Tera-MIND can currently infer. In comparison, we explored an additional marker, *Kcnj8*, which encodes the ATP-sensitive potassium (K-ATP) channel and modulates brain vascular smooth muscle development^9^. In the adult mouse brain, *Kcnj8* is predominantly expressed in pericytes and venous vascular smooth muscle cells. As shown in the side-by-side comparison in Fig. 4, vascular reconstruction differs markedly when *Cldn5* is replaced with *Kcnj8*. This pattern was consistently observed across experiments, and *Cldn5* and *Acta2* generally remain the optimal candidates for visualizing a broader spectrum of vascular structures. Future spatial transcriptomic datasets with richer vascular panels, for example including genes such as *Pdgfrb* (pericytes) or *Slc2a1* (endothelial cells), could enable more detailed modeling of cerebrovascular organization and vascular compartmentalization.

Guided by the spatial co-expression of vasculature-relevant genes *Cldn5* and *Acta2*, we demonstrated the utility of generative modeling Tera-MIND for vasculature representation using spatial transcriptomics data. This multi-modal approach provides an ethically responsible and cost-effective platform for probing vascular heterogeneity in silico and can thereby enable systematic, high-throughput analysis of vascular organization across diverse biological contexts.

## Materials and methods

### Tera-MIND generative framework

The Tera-MIND model is a novel generative framework designed for tera-scale biomedical data. It leverages paired spatial transcriptomics arrays and co-registered histological images to simulate mouse brains in space. Tera-MIND employs a patch-wise and boundary-aware training strategy, enabling the model to learn from smaller sub-volumes. This design allows for efficient training and inference on high-volume datasets, making whole-brain modeling at cell-level resolution computationally feasible under relatively mild hardware requirements^3^. A summary of the generative framework is provided in supplementary methods, with extended methodological details described in Wu et al.^3^

### Spatial transcriptomics data

Recent efforts by a network of researchers has resulted in the generation of a detailed cellular atlas of the mouse brain^7,10–12^. The mouse brain data set of coronal sections with high-resolution images of DAPI (4’,6-diamidino-2-phenylindole) and Poly-T (mRNA) stained tissue with the corresponding spatially indexed transcriptomic readout used for development of Tera-MIND was published by Yao et al.^7^. Training of Tera-MIND on this data set is described in more detail in the previous study^3^. In short, by training a patch-based and boundary-aware diffusion model on gene expression input and corresponding histological images, it predicts the spatial associations between molecular gene expression markers and histological image patterns.

As described previously^3^, data sets for three different mouse brains were available^7,10^. The primary generation results are based on the data set of a female mouse brain (P56), and the other two available data sets (one female and one male mouse brain, P56) were used as training data. We refer interested readers to the previous study^3^ for a more detailed comparison of ground truth and generated images. Here, we include one example of a ground truth (Fig. 1c) and a corresponding generated (Fig. 1d) DAPI image of a coronal mouse brain section.

### Validation and analysis

After training the model based on the two training data sets (mouse brain ID 609882 and 609889) with corresponding transcriptomic and histological data, the model of the brain vasculature based on *Acta2* and *Cldn5* expression was generated on the unseen third data set (mouse brain ID 638850) (Fig. 1a and b, model training and test). The modeled vasculature was validated against available imaging data and known anatomical landmarks from the Xiong et al. study^6^.

## Supplementary Information

Below is the link to the electronic supplementary material.

Supplementary Material 1

## Data Availability

Spatial transcriptomic and histological data (DAPI and Poly-T) for all three mouse brain atlases can be accessed via 10.35077/g.610. The processed spatial transcriptomic and histological data can be accessed via 10.35077/g.1176. The code and details on model training and testing are made available at the repository github.com/CTPLab/Tera-MIND.

## Declarations

### Data visualization and figure assembly

Figure illustrations were created and assembled in *Numbers* (version 14.1) and *Adobe Illustrator* (version 29.4).
