# Supplementary material for: Generative cerebral vasculature visualization using spatial transcriptomic data
Source: Sci Rep. 2026 Mar 31;16:15540. doi: 10.1038/s41598-026-46455-4 (PMC13187267; doi:10.1038/s41598-026-46455-4)
Supplement: Supplementary file 1 — Supplementary Material 1 [file 41598_2026_46455_MOESM1_ESM.docx]

Generative cerebral vasculature visualization using spatial transcriptomic data

Ingrid Berg^1^, Jiqing Wu^2,^*, Viktor H. Koelzer^2,3,^*

^1^Department of Pathology and Molecular Pathology, University Hospital Zurich, University of Zurich, Switzerland

^2^Department of Biomedical Engineering, University of Basel, Switzerland

^3^Institute of Medical Genetics and Pathology, University Hospital Basel, Switzerland

*Corresponding Authors: Jiqing Wu, Viktor H. Koelzer

**Email:**  [Jiqing.Wu@unibas.ch](mailto:Jiqing.Wu@unibas.ch), [Viktor.Koelzer@usb.ch](mailto:Viktor.Koelzer@usb.ch)

**Classification:** Major: Biological Sciences, Minor: Neuroscience

**Keywords:** Spatial transcriptomics, generative modeling, cerebral vasculature

**Supplementary Methods**

Tera-MIND generative framework for vascular transcriptomic modeling

Tera-MIND is a generative framework that integrates spatial transcriptomics arrays with co-registered histological images to model brain structures at cellular resolution^1^. In contrast to approaches that detect individual marker genes, Tera-MIND enables the generation of spatial vascular maps by learning spatial associations between gene expression and histological image patterns across entire tissue volumes.

Considering spatial mRNA readouts as three-dimensional “images”, a patch-based diffusion model was applied for efficient training on tera-scale volumetric data, while a boundary-aware denoising component ensured smooth transitions between adjacent patches. Central to the framework is a three-dimensional self-attention block for spatial *gene*-*gene* interactions, which captures correlations among vascular marker genes such as *Cldn5* and *Acta2*. In this process, spatial mRNA counts are first converted into three-dimensional gene-expression arrays and passed through the gene-gene self-attention module, which learns patterns of gene co-occurrence within shared spatial neighborhoods and encodes them into a latent molecular context for each location. Subsequently, this context conditions the UNet-based denoising network, allowing the model to rely not only on isolated mRNA counts but also on the learned joint patterns of vascular and spatially informative genes. In the present application, this enables the model to use combined *Cldn5* and *Acta2* expression patterns to guide the synthesis of vascular-like patterns from sparse spatial transcriptomic input. For a more informative illustration, we refer readers to the video demonstrations on the project webpage https://musikisomorphie.github.io/Tera-MIND.html

Training was performed on publicly available mouse brain atlases comprising paired spatial transcriptomics and histological data^2^. Two P56 mouse brains (male and female) were used for model training, and a third unseen P56 female brain served as the held-out test data. This ensured reproducibility and generalization across individuals. Quantitative evaluations against ground truth images were reported in the prior study^1^ and showed high-fidelity modeling across multiple scales, with minimal discrepancies measured on cellular features such as nuclear size and cell number^1^.

Despite the tera-scale size of the data (~0.77 teravoxels), inference is computationally efficient: whole-brain models can be generated in a patch-wise manner within about a week on a single DGX system equipped with A100 GPUs. Further, the *Cldn5*-*Acta2* guided vasculature mapping takes less than 4 hours.

By seamlessly generating a transcriptomic field rather than classifying isolated spots, Tera-MIND produces coherent vascular hierarchies, from endothelial capillaries to smooth muscle-associated arterial structures. This property enables the visualization of vascular continuity and the analysis of gene-gene interactions within the cerebrovascular network.

For comprehensive architectural, training, and quantitative evaluation details of the underlying

Tera-MIND framework, readers are referred to Wu *et al.*^1^, where model performance and comparisons to baseline methods were assessed extensively.

**References**

1. Wu, J., Berg, I., Li, Y., Konukoglu, E. & Koelzer, V. H. Tera-MIND: Tera-scale mouse brain simulation via spatial mRNA-guided diffusion. Preprint at https://doi.org/10.48550/ARXIV.2503.01220 (2025).

2. Yao, Z. *et al.* A high-resolution transcriptomic and spatial atlas of cell types in the whole mouse brain. *Nature* **624**, 317–332 (2023).

**Supplementary Figure 1**

**
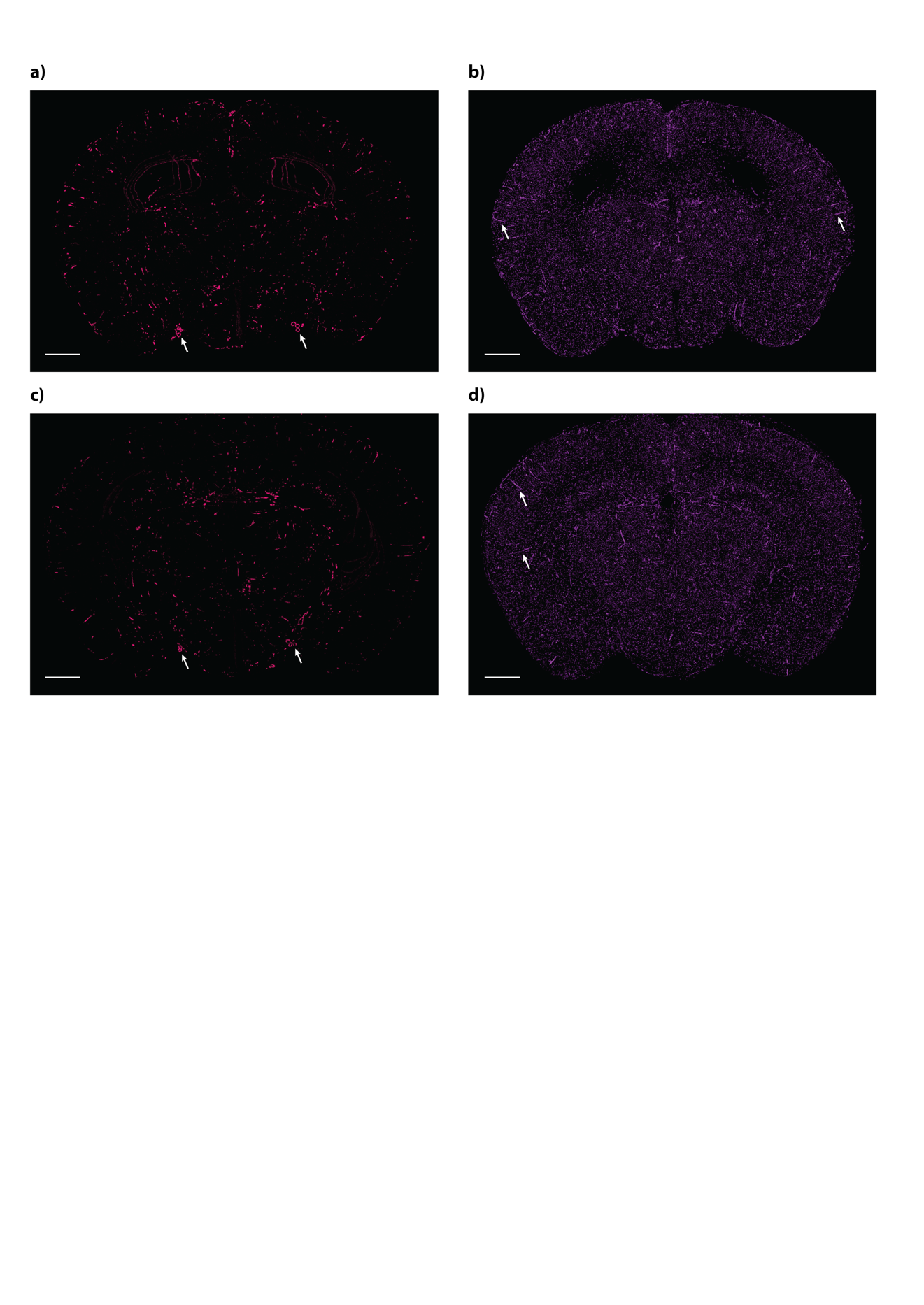
**

**Supplementary Figure 1. Additional illustrations of generative modeling of cerebrovascular architecture from spatial transcriptomic data. a-d)** Generated *Acta2* (**a**, **c**) and *Cldn5* (**b**, **d**) expression patterns, visualized in an overlay of 4 consecutive coronal sections. These results, from the two mouse brain data sets that were used for model training, demonstrate reproducibility of vascular-like anatomy (indicated with white arrows) across individuals. **a)** and **b)** represent one (ID: 609889), **c)** and **d)** represent the other (ID: 609882) mouse brain data set. **a-d)** Length of scale bar (white, bottom left corner) is 1mm.
